# *Streptococcus gallolyticus* abrogates anti-carcinogenic properties of tannic acid on low-passage colorectal carcinomas

**DOI:** 10.1038/s41598-020-61458-5

**Published:** 2020-03-13

**Authors:** Sonja Oehmcke-Hecht, Vanessa Mandl, Lukas Tim Naatz, Lara Dühring, Juliane Köhler, Bernd Kreikemeyer, Claudia Maletzki

**Affiliations:** 1 Institute of Medical Microbiology, Virology and Hygiene, Rostock University Medical Center, Rostock, Germany; 2 Department of Internal Medicine, Medical Clinic III - Hematology, Oncology, Palliative Care, Rostock University Medical Center, Rostock, Germany

**Keywords:** Cancer, Gastrointestinal cancer

## Abstract

The tannase-producing Gram-positive bacterial species *Streptococcus gallolyticus* subsp. *gallolyticus* (*Sgg*) is an opportunistic pathogen of the human gut and strongly associated with colorectal cancer (CRC). A unique feature of *Sgg* is its ability to degrade tannic acids (TA). TA constitute an important part of the human diet with known anti-tumorigenic properties. Here, we examined whether *Sgg* is able to protect tumor cells from the toxic effect of TA and thus drive tumorigenesis indirectly. Human CRC cell lines (n = 8) were treated with increasing concentrations of TA. We confirmed the cytotoxic activity of TA in a dose-dependent manner. In virtually all cell lines, viability decreased significantly (>60% inhibition). Moreover, pyrogallol, the degradation product of TA, had no effect on the tested cell lines. This suggests a specific effect of TA. Cytotoxicity was due to necrosis and induction of senescence in residual cells. Finally, when TA was degraded by *Sgg*, the cytotoxic effect could be abolished. Tumor cells even responded with boosted cell proliferation, highlighting the impact of *Sgg* on CRC progression. We here provide another piece of evidence for the active interplay between *Sgg* and cancer preventive components. These data will help to move forward in designing concepts for therapeutic and eventually also prophylactic approaches to combat gastrointestinal malignancies.

## Introduction

The opportunistic pathobiont *Streptococcus gallolyticus subsp. gallolyticus (Sgg)*, previously known as *Streptococcus bovis* biotype I, is asymptomatically found in the gastrointestinal tract of humans (2.5–15%), ruminants and birds. The bacteria belong to the *Streptococcus bovis/Streptococcus equinus* complex (SBSEC)^1^, a highly diverse bacterial group of Gram-positive, non-hemolytic Lancefield group D commensals. The original division of the SBSEC into *S. bovis* and *S. equinus* has further changed over the past years, reaching the current splitting into seven main (sub)species, *Streptococcus infantarius* subsp. *infantarius* (*Sii*), *Streptococcus lutetiensis*, *Streptococcus gallolyticus* subsp. *pasteurianus* (*Sgp*), *Streptococcus gallolyticus* subsp. *macedonicus* (*Sgm*), *Streptococcus gallolyticus* subsp. *gallolyticus* (*Sgg*), *Streptococcus alactolyticus* and *S. equinus*^1–3^. *Sgg* is estimated to be the causative agent of endocarditis in 11–14% of cases^4^. Interestingly, multiple studies have shown that endocarditis due to *Sgg* is often associated with gastrointestinal malignancy^5^. Colonization of the gut by *Sgg* is related to the occurrence of colorectal cancer (CRC), the presence of *Sgg* in CRC patients with up to 74% is much higher than in healthy people^6,7^. It has been suggested that *Sgg* itself may play a causal role in CRC development, as the bacteria promote tumor proliferation by upregulating β-catenin^8^.

On the other hand, it was previously shown that CRC-specific conditions – namely the increased concentration of bile acids – promote *Sgg* colonization, as secondary bile acids strongly enhanced activity of a bacteriocin (gallocin) that is produced by the bacteria^9^. Further on, *Sgg* is remarkably resistant to bile acids^10^. Another distinctive characteristic of *Sgg* is its ability to degrade tannic acids (also named “gallotannin”), a property, which led this bacterium to be named “gallolyticus”^11^. Tannic acid (TA) belongs to the family of hydrolysable tannins. It is found in a variety of fruits and beverages, such as tea, coffee and red wine, thus TA constitutes an important part of the human diet^12,13^. Moreover, TA inhibits the proliferation of diverse tumor cells, including CRC, without being toxic to normal cells^14,15^. TA constrains the expression of inflammatory genes and cytokines in human lung cancer cells^16^, and has anti-metastatic potential^17^. Finally, it was shown recently that TA inhibits telomerase activity *in vitro* and *in vivo*^18–20^.

Intriguingly, especially bacteria that selectively colonize tumorous tissues in CRC patients - but not adjacent non-malignant tissues - contain tannase homologous genes^21^, which allows them to degrade TA. In contrast, the closely related (sub)species *Streptococcus infantarius* subsp. *infantarius (Sii)* have lost genes for detoxifying toxic substances such as TA^22^. *Sgg* hydrolyze TA by tannase into gallic acid. Gallic acid will be decarboxylated by a gallate decarboxylase to pyrogallol (PG)^23^. Of note, Jiménez *et al*. considered *Sgg* as the best bacterial cellular factories for (gallo)tannin degradation so far known^24^.

We therefore hypothesized whether this special feature of *Sgg* might protect the tumor cells from the toxic effect of TA. We could show that all of the investigated low passaged CRC cell lines are susceptible towards TA treatment. Moreover, PG, the degradation product of TA, had no cytotoxic effect on the tested cell lines. Finally, when TA was degraded by *Sgg*, the toxic effect on the tumor cells could be abolished. This supports the assumption that colonization of the tumor by *Sgg* protects the tumor cells against otherwise toxic plant components that will be consumed by a normal human diet.

## Methods

### Tumor cell lines and culture media

CRC cell lines HROC24 T1 M1, HROC60, HROC173, HROC183 T0 M2, HROC257 T0 M1, HROC285 T0 M2, HROC324, and HROC370 were described before and obtained from Cell line services (Eppelheim, Germany)^25^. The CRC cell lines HT29 and CaCo2 were originally obtained from the German collection of cell cultures (DSMZ; Braunschweig, Germany) and routinely cultured in our lab. Cells were maintained in full medium: DMEM/HamsF12 supplemented with 10% fetal calf serum (FCS), glutamine (2 mmol/L) and antibiotics (medium and supplements were purchased from PAA, Cölbe, Germany).

### Bacterial strain and culture conditions

*Sgg* UCN34 was isolated at the Hospital in Caen (Calvados, France)^26^. *Sii* JIM 9407 was isolated from human origin in Africa^27^. Bacteria were grown on blood agar plates at 37 °C, aerobically, overnight and subsequently stored at 4 °C. For further use, overnight cultures were cultivated in BHI broth at 37 °C under a 5% CO_2_–20% O_2_ atmosphere in the presence or absence of TA (Sigma Aldrich, Germany). The optical density was determined in a Spectramax (Molecular Devices, USA) or the bacteria were plated every hour to determine the CFU/ml.

### Treatment protocol

CRC cell lines (8 in total) were treated with increasing TA or PG concentrations (ranging from 2.5 to 100 µM) for 72 h. Thereafter, selected cell lines were used for different experiments. Bacterial supernatants from TA degradation experiments were diluted 1:10 in cell culture medium before applied to the cells.

### Flow cytometric DNA analysis and crystal violet staining

Cells were grown to about 70% confluence in 24-well culture plates and then incubated in complete medium with or without TA and PG for 72 hours, respectively. Subsequently, cells were prepared for crystal violet staining, as well as flow cytometric apoptosis and cell cycle analysis as reported previously^28^. Additionally, a Yo-Pro-1/PI-based assay for discriminating (early) apoptotic and necrotic cells was applied as described before^25^.

### Wound healing assay

Tumor cell proliferation after TA or PG treatment was investigated as described before^29^ Briefly, cells were seeded in 6-well plates and grown to 100% confluency. Monolayers were scratched with a 20 μL pipette tip to induce a wound. Wounded edges were imaged using a Zeiss inverted microscope. Images were taken daily for a period of 5 d under a × 10 objective lens.

### Measurement of cellular senescence

Induction of senescence after 5 days of treatment with TA or PG was measured by β-galactosidase, detectable at pH 6.0 with the artificial substrate X-gal^30^.

### Determination of tannase activity

Tannase activity of *Sgg* UCN34 or *Sii* was determined by a spectrophotometric method^31^. Briefly, overnight cultures of bacteria were set to 10^8^ CFU/ml in PBS and incubated with 2 mM methylgallate for 24 h at 37 °C. After 24 h of incubation, 0.5 ml of the suspension were alkalinized with equal amounts of saturated NaHCO_3_ solution (pH 8.6) and then left in the atmosphere at room temperature for 60 min. This alkalization facilitated nonenzymatic oxidation of gallic acid (released by tannase from methylgallate) to form polymerized compounds of o-quinone derivatives, resulting in green to brown coloration of the medium. Absorption was measured at 440 nm.

### Degradation of TA by *Sgg or Sii*

Overnight cultures of bacteria were set to 10^8^ CFU/ml in PBS and incubated with 1 mM TA at 37 °C for 24–48 h. As control TA solution (1 mM) without bacteria was used. Absorption of TA was measured at 310 nm. Bacterial supernatants for incubations on cells were sterilfiltered and stored at −20 °C until use.

### Statistics

All values are reported as mean ± SD. After proving the assumption of normality, differences between controls and treated cells were determined by using the unpaired Student’s *t*-test. If normality failed, the non-parametric Mann-Whitney *U*-Test was applied. In case of multiple comparisons, one way ANOVA on ranks (Bonferroni’s Multiple Comparison Test or Holm-Šídák) was applied. Statistical evaluation was performed using GraphPad PRISM software, version 5.02 (GraphPad Software, San Diego, CA, USA). The criterion for significance was taken to be p < 0.05.

## Results

### Growth inhibition of low passage CRC cell lines by TA

**Figure 1:**
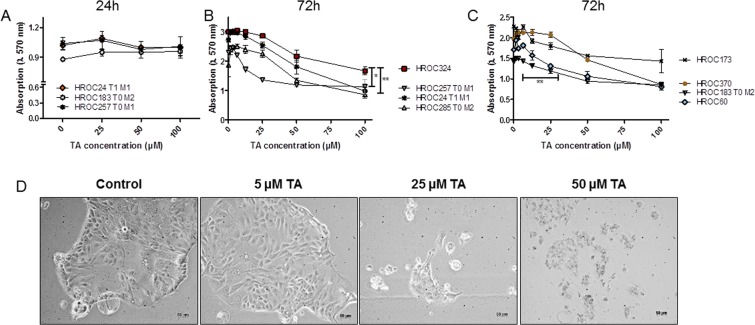
Crystal violet staining and morphology of cell lines treated with increasing doses of TA. (**A–C**) Quantitative analysis of adherent cells upon (**A**) 24 hours and (**B,C**) 72 hours of treatment. Untreated cells were used as controls. TA reduced cell growth of different patient-derived CRC cell lines in a dose-dependent manner. Results show data of three independent experiments. Mean ± SD. **p = 0.0065 HROC257 T1 M1 vs. HROC324; p = 0.0259 HROC285 T0 M2 vs. HROC324, **p = 0.0071 HROC183 T0 M2 vs. HROC370; two-tailed t-test. **(D)** Light microscopy of HROC285 T0 M2 cells after 72 h treatment with increasing TA concentrations. 1 × 10^4^ cells were seeded and after overnight incubation treated with different concentrations of TA. Pictures were taken 72 h after treatment.

To investigate the susceptibility of CRC cell lines against TA, 8 patient-derived low passage CRC cell lines were treated with increasing TA concentrations. Following 24, 48, and 72 hours of incubation, biomass was quantified by crystal violet staining (Fig. 1A–C). TA reduced the biomass time-dependently with significant inhibition after 72 hours at a concentration of 12.5 µM (Fig. 1B,C). Of note, minor differences were observed between individual cell lines.

**Figure 2:**
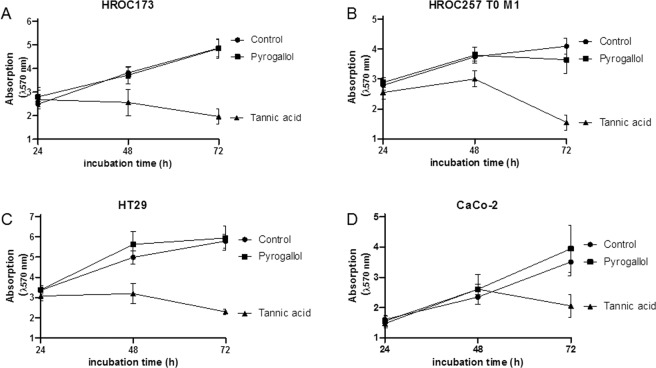
Crystal violet staining of different cell lines treated with 100 µM TA or PG. TA, but not its metabolite PG, reduced cell growth. Quantitative analysis of adherent cells was done by crystal violet staining after 24, 48 and 72 h of incubation. Untreated cells (control) were used as controls. Results show data of three independent experiments. Mean ± SEM.

Microscopic observation confirmed these data with a destroyed cell layer and dead cells after 72 h of incubation with 25 µM TA (Fig. 1D shows exemplary images of HROC285 T0 M2 cells). PG is a degradation product of TA, with potential cytotoxic activity. However, even at high concentrations (100 µM), no significant reduction of biomass was measured in different cell lines (Fig. 2). Of note, no differences were seen between low (HROC173, HROC257 T0 M1) and high passage (HT29, CaCo-2) cells (Fig. 2).

### TA induces predominantly necrosis in target cells

**Figure 3:**
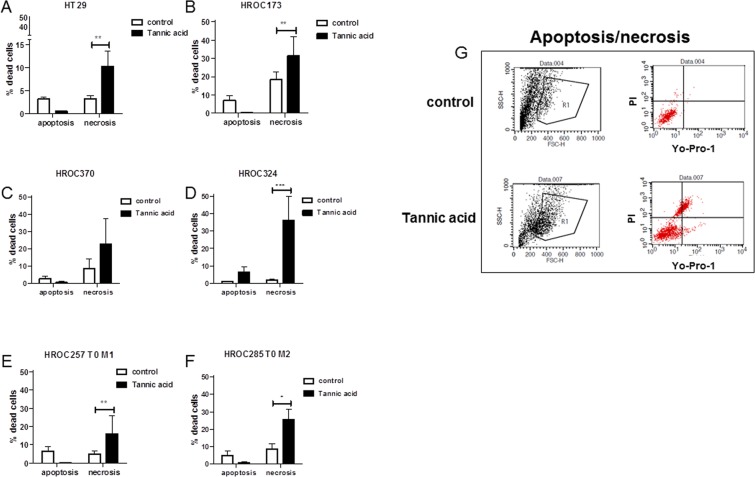
Apoptosis and necrosis measured after treatment with TA. 1 × 10^4^ cells were seeded and after overnight incubation treated with 100 µM TA. Apoptosis and necrosis was determined via flow cytometry 48 h after treatment. (**A**–**F**) Results show data of three independent experiments. Mean ± SD, *p < 0.05, **p < 0.0065, ***p = 0.0006; two-tailed t-test (**G**) Representative flow cytometry dot blots of HROC173 cells either being untreated (control, upper panel) or treated with TA (lower panel).

**Figure 4:**
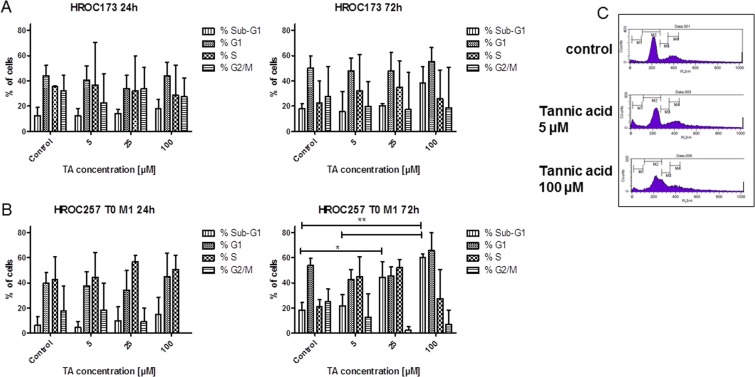
Cell cycle analysis after treatment with TA. 1 × 10^4^ cells of **(A)** HROC173 and **(B)** HROC257 T0 M1 were seeded and after overnight incubation treated with increasing TA concentrations. Cell cycle phases as well as numbers of cells with Sub-G1 DNA were examined at 24 and 72 h after treatment. Residual cells were mainly arrested in G1 or G2/M-phase. Results show data of three independent experiments. Mean ± SD, *p < 0.05; **p < 0.01; one way ANOVA on ranks (Bonferroni’s Multiple Comparison Test). (**C**) Representative flow cytometry blots of HROC173 cells either being untreated (control, upper panel) or treated with TA (middle and lower panel) for 72 hours.

To improve our understanding on the type of cell death induced by TA, 6 CRC cell lines (5 low and 1 high passage) were subjected to flow cytometric apoptosis/necrosis analysis. We observed predominantly necrosis in all cell lines (Fig. 3A–F; 3G: exemplary results of HROC173 cells). Levels of necrotic tumor cells, as defined by positive PI staining, increased significantly by about 10–40% in the different cell lines after treatment with TA (Fig. 3). Late apoptotic cells, being Yo-Pro-1^+^/PI^+^ were below 10% in the controls, and additionally reduced after incubation with TA in 5/6 cell lines. The only exception was seen in HROC324; showing increased numbers of apoptotic cells (Fig. 3D). Complementary examination of cell cycle principally confirmed these findings. While most of the cells showed elevated levels of sub-G1 DNA (up to 50%; HROC257 T0 M1 72 h: p < 0.01 TA 100 µM vs. ctrl and vs. TA 5 µM; p < 0.05 TA 25 µM vs. ctrl), residual cells were either arrested in G1 or mainly in G2/M-phase (Fig. 4).

### Influence of TA and PG on senescence, invasion and migration

**Figure 5:**
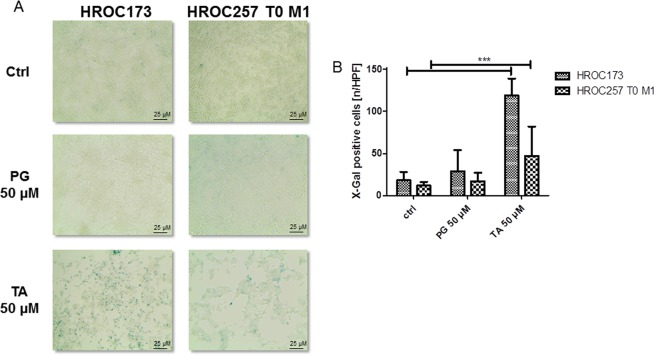
Detection of senescence in HROC173 and HROC257 T0 M1 cells after incubation with 50 µM TA. 1 × 10^4^ cells were seeded and after overnight incubation treated with 50 µM TA or PG for 5 days. Senescent cells were detected by blue color after beta-galactosidase assay. Treated cell show increased numbers of beta-galactosidase-positive cells. (**A**) Representative light microscopic images. Original magnification 10x. (**B**) X-Gal quantification. HPF – high power field; Mean ± SD, ***p < 0.0001; two-tailed t-test.

TA was previously described to induce senescence in the CRC cell line HCT116^32^. Thus, we assessed the potential of TA to induce senescence in four selected cell lines (low passage: HROC173, HROC257 T0 M1; high passage: HT29, CaCo-2) after 5 days of incubation with 50 µM TA or PG. For all cell lines, induction of senescence was observed. Figure 5A shows exemplary images of HROC173 and HROC257 T0 M1, along with quantitative analysis depicted in Fig. 5B. As expected, PG had no effect on the cells (Fig. 5, middle panel), while TA led to a significant increase in senescent cells (p < 0.0001 vs. ctrl, Fig. 5B).

**Figure 6:**
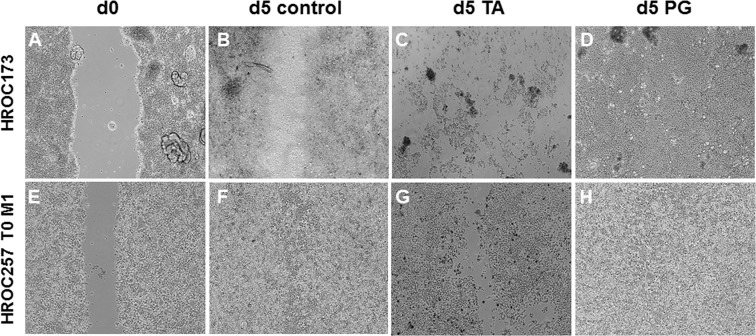
Wound healing assays were performed with HROC173 and HROC257 T0 M1 cells. Representative light microscopic images of migrating tumor cells at day 0 (d0) and day 5 (d5) following incubation with 50 µM TA or PG. Original magnification 10x.

Next, a wound healing assay was used to examine proliferation in the presence of TA or PG (Fig. 6). Representative pictures of low passage cell lines HROC173 (Fig. 6A–D) and HROC257 T0 M1 (Fig. 6E–H) are given. While control cells proliferated and closed the wound within 5 days (Fig. 6B,F), cells treated with 50 µM TA did not proliferate, the cell layer was destroyed and remaining cells looked unhealthy (Fig. 6C,G). PG had no inhibiting effect on proliferation, the wound was fully closed within 5 days and the cells appeared normal (Fig. 6D,H). We further investigated whether TA or PG might influence the invasive behavior of CRC cells; however, neither substance had a significant impact on invasion (*data not shown*).

Taken together, TA, but not its degradation product PG, is toxic on CRC cell lines and induces senescence in residual cells.

### *S. gallolyticus* UCN34, but not *Sii*, degrades TA

**Figure 7:**
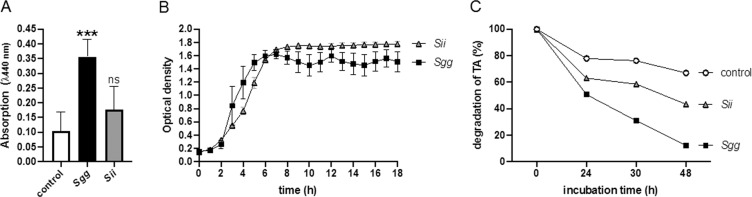
TA degradation and growth of bacteria in the presence of TA (A) Overnight cultures of bacteria (10^8^ CFU/ml) were incubated with 2 mM methylgallate for 24 h at 37 °C. Buffer without bacteria were used as control. Gallic acid (released by tannase from methylgallate) was oxidized and measured at 440 nm. **(B)** Growth curve of *Sgg or Sii* in cell culture medium containing 1000 µM TA. The optical density was determined in a Spectramax **(C)** Overnight cultures of bacteria (10^8^ CFU/ml) or buffer (control) were incubated in 1000 µM TA for up to 48 h. Absorption of the original solution was set to 100%. Mean ± SD, ***p < 0.0005; two-tailed t-test.

The strain *Sgg* UCN34 contains two genes for tannase (GALLO_1609 and GALLO_0933) as well as two decarboxylase genes^10^, important for TA degradation. We confirmed tannase activity of *Sgg* UCN34 by a spectrophotometric method^33^, using the *Sii* strain as a negative control. This method uses methyl gallate as substrate degraded by bacterial tannase, whereby gallic acid will be released and detected at A440 nm. Figure 7A shows that *Sgg*, but not *Sii*, produced significant higher amounts of gallic acid, comparing to control samples without bacteria.

TA was described to exert strong antimicrobial activity against bacteria without tannase, thus growth of *Sgg* and *Sii* in the presence of high TA concentrations was tested. However, TA had no influence on *Sii* or *Sgg* growth at concentrations of 1000 µM (Fig. 7B).

TA in solution can also be detected at 310 nm^34^. When bacteria were incubated in a 1000 µM TA solution, a decrease of absorption (about 50%) could be detected after 24 hours of incubation with *Sgg* (Fig. 7C). After 48 h the TA content was reduced to 15% in the presence of *Sgg*, and to 43% in the presence of *Sii*, whereby in the presence of buffer the TA content was reduced to 67% (Fig. 7C). These data indicate that the TA-solution is relatively stable in the absence of bacteria, and actively degraded by *Sgg*.

### Degradation of TA by *Sgg* obliterated its toxic effect on tumor cells

**Figure 8:**
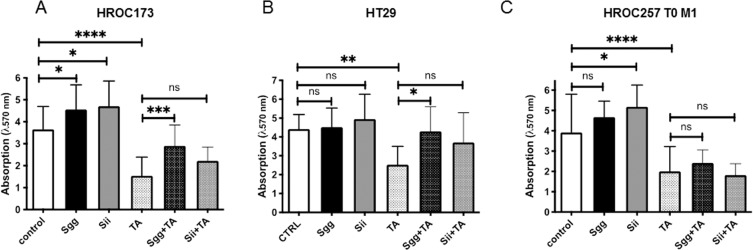
Growth of CRC cells after incubation with bacterial supernatants. TA was incubated with bacteria (*Sgg* + TA, *Sii* + TA) or buffer (TA), for 24 h to reduce the TA concentration in the solution. Bacteria without TA (*Sgg, Sii*) were used as negative control. Cells treated with buffer alone were used as control. Bacterial supernatants were sterilized by filtration and added to three different tumor cell lines at final estimated concentration of 100 µM TA. Quantitative analysis of adherent cells was done by crystal violet staining after 72 h of incubation. Results show data of three independent experiments. Mean ± SD; *p < 0.05, **p < 0.005, ***p < 0.0005, ****p < 0.0001; one way ANOVA (Holm-Šídák test).

As TA can be degraded by *Sgg* we next tested whether this might protect CRC cells from killing. To address this experimentally, TA was incubated with *Sgg*, *Sii* or buffer, to reduce the TA concentration by the bacteria. After incubation, bacterial supernatants were sterilized by filtration and added to different tumor cell lines. TA alone was used as a positive control for the toxic effect. Additionally, bacterial supernatants without TA served as controls. CRC biomass was quantified by crystal violet staining after 72 h of incubation. As shown before, treatment with 100 µM TA significantly reduced the biomass in all cell lines (Fig. 8). However, this effect was partially abolished when the TA solution was pretreated with *Sgg* bacteria. This effect was not observed after pretreatment with *Sii* bacteria (Fig. 8). Intriguingly, bacterial supernatant (without TA) from both species stimulated growth of the HROC173 cell line significantly (Fig. 8A). These data additionally suggest a tumor growth-promoting effect of bacterial compounds. Taken together, degradation of TA by *Sgg* bacteria abrogates its toxic effect on two tumor cell lines.

## Discussion

Tannic acids (TA) widely occur in common food such as berries, grapes, mangoes, and nuts, but also in a variety of beverages such as coffee, tea or red wine for a review see^35^. TA are additionally extensively used as clarifying and refining agent in food. Thus, a daily intake of sufficient amounts of TA by most of the humans is very likely.

In an experimental mouse model, it has been demonstrated that very low dose dietary TA administration prevented animals against spontaneous hepatic neoplasm development. The TA amount consumed daily by each mouse in this study corresponded to the human situation, and further support the strong anti-carcinogenic effect of TA^36^.

In our study, we examined the direct effects of TA on a panel of patient-derived as well as long-term cultured CRC cell lines. TA induced a biphasic, but dose-dependent growth inhibiting effect in all CRC cell lines tested, supporting recent findings in which TA was described to exert anti-proliferative activity on numerous cancers. TA affected migratory potential in all cell lines, and cytotoxicity was mainly due to necrosis with additional senescence induction in residual cells. Mechanistically, this is likely attributable to local amino acid starvation that triggers cancer cell senescence. This effect is well-described for other amino-acid degrading enzymes, such as the bacterial arginine deiminase^29,37^. TA increases the production of reactive oxygen species by altering the redox balance in the cell. This finally induces cell cycle arrest as described for HCT116 cells^38^, supported by our own observations.

TA is thus considered a potential anticancer agent^39–41^, with additional ability to act as chemosensitizer^42^. The latter is a characteristic quite common for other polyphenols as well. The selective antitumoral efficacy of TA was further shown here by lacking cytotoxicity of PG, a degradation product of TA. While this substance was found to exert growth-inhibiting and pro-apoptotic against breast cancer cells *in vitro*, viability as well as migratory activity of CRC was not substantially affected^43^. Moreover, the natural concentration of PG in fruits and vegetables is generally low, but likely increases by tannin-degrading bacteria, such as *Sgg*^21^.

Apart from the therapeutic effect of TA, a recent study even proposed this substance as a promising pyruvate kinase isoenzyme M2 inhibitor for CRC prevention^39^. Still, clinical evidence of dietary polyphenols as chemopreventive compounds is scarce and at least partially attributable to the fact that TA concentrations in the gastrointestinal tract are hardly measurable and backtracked to mediate a direct tumor growth inhibitory effect.

TA possess numerous pharmacological and thus biologically relevant characteristics, such as anti-inflammatory and bacteriostatic activity with MIC values between 0.012 and 1 g/l^44^. Here, we show that *Sgg*, as well as *Sii* are able to grow in the presence of high TA concentrations (1000 µM ≜ 1701 g/l). However, our data indicate that only *Sgg* actively degrades TA, supporting earlier reports^45^. Physiologically, this characteristic is considered an adaptive mechanism to withstand stress conditions associated with the presence of these phenolic compounds.

While TA may have potent antitumoral activity, *Sgg* is a common and selective gut colonizer of CRC patients. This high prevalence of *Sgg* in CRC patients - compared to healthy persons - might be associated with specific conditions created by the tumor, such as increased concentration of bile acids and a slowed food flow. This leads to accumulation of plant-derived fiber carbohydrates and possibly TA, which may represent a favorable microenvironment for establishment and proliferation of *Sgg*^10^.

Whether *Sgg* is a passenger or a driver bacterium has not been completely elucidated, but is just beginning to become clear. Pasquereau-Kotula described a two-hypothesis model, by which *Sgg* either contributes to CRC development as a consequence of local microbial imbalance or actively accelerates transformation due to high colonization in pre-malignant epithelium along with specific inflammatory responses and increased cell proliferation^46^. Growing evidence supports the former with *Sgg* not being a bona fide pathogen, but likely benefits from the microenvironment created by preneoplastic glands in the gut^9^. A recent study supports these findings by identifying different interactions of *Sgg* strains with human colon cancer cells. This finding was confirmed *in vivo* using the azoxymethane-induced CRC model^47^. The authors suggested a close contact between *Sgg* and host cells to be important for mediating growth promoting effects. Our study adds a piece to this and thus broadens our current knowledge on the interaction of *Sgg* with CRC.

Yet, while some gastrointestinal microbes were described to preferentially degrade hydrolyzable tannins (e.g. *Lactobacillus plantarum*, *Lonepinella koalarum* and *Selenomonas ruminantium, Staphylococcus lugdunensis*) almost none of them have been linked to CRC. A recent study identified an active tannase enzyme from the oral pathogen *Fusobacterium nucleatum*^48^ – only lately described to promote CRC similar to *Sgg*. Of note, serum anti-Fn antibody IgA combined with carbohydrate antigen 19-9 and carcinoembryonic antigen are reliable screening tools and just as for *F. nucleatum*^49^, efforts to use *Sgg* antibodies as serum biomarkers are on the way for CRC risk stratification^50^. Hence, such microbial–based approaches will facilitate diagnostic efficacies prospectively.

Summarizing our findings, this study hypothesizes that colonization of CRC by *Sgg* protects tumor cells against the cytotoxic effects of TA. Detection and elimination of these bacteria – and certainly other TA-degrading microbes – might finally support effective treatment of CRC. Further *in vivo* studies are necessary to proof this hypothesis.

## Conclusions

This study confirms (I) the cytotoxic activity of tannic acids against colorectal cancer cells and (II) describes a protective role of the tannic acid-degrading bacteria *Sgg* most likely providing a growth advantage of colorectal cancer cells. Hence, we here add another piece of evidence for the relevance of the bacterial microenvironment in cancer initiation and most likely also progression. This study constitutes a fundamental basis for upcoming preclinical research to diagnose and on the long run combat cancer.

## Data Availability

The datasets used and/or analyzed during the current study are available from the corresponding author on reasonable request.
